# Use of Prostaglandin E1 in the Management of Congenital Diaphragmatic Hernia–A Review

**DOI:** 10.3389/fped.2022.911588

**Published:** 2022-07-01

**Authors:** Srirupa Hari Gopal, Neil Patel, Caraciolo J. Fernandes

**Affiliations:** ^1^Section of Neonatology, Department of Pediatrics, Baylor College of Medicine, Houston, TX, United States; ^2^Department of Neonatology, Royal Hospital for Children, Glasgow, United Kingdom

**Keywords:** Congenital Diaphragmatic Hernia (CDH), pulmonary hypertension, prostagladin E1, Patent Ductus Arteriosus (PDA), ventricular dysfunction

## Abstract

Congenital diaphragmatic hernia (CDH) is a rare congenital anomaly, whose presentation is complicated by pulmonary hypertension (PH), pulmonary hypoplasia, and myocardial dysfunction, each of which have significant impact on short-term clinical management and long-term outcomes. Despite many advances in therapy and surgical technique, optimal CDH management remains a topic of debate, due to the variable presentation, complex pathophysiology, and continued impact on morbidity and mortality. One of the more recent management strategies is the use of prostaglandin E1 (PGE1) infusion in the management of PH associated with CDH. PGE1 is widely used in the NICU in critical congenital cardiac disease to maintain ductal patency and facilitate pulmonary and systemic blood flow. In a related paradigm, PGE1 infusion has been used in situations of supra-systemic right ventricular pressures, including CDH, with the therapeutic intent to maintain ductal patency as a “pressure relief valve” to reduce the effective afterload on the right ventricle (RV), optimize cardiac function and support pulmonary and systemic blood flow. This paper reviews the current evidence for use of PGE1 in the CDH population and the opportunities for future investigations.

## Introduction

Congenital diaphragmatic hernia (CDH) is a rare anomaly, characterized by a defect in the diaphragm causing abdominal contents to protrude into the thoracic cavity. The incidence of CDH is 1 in 2,500 to 1 in 3,500 live births (1). It occurs 70%−75% of the time in the posterolateral aspect of the diaphragm, with over 85% occurring on the left side (2). CDH can also be associated with congenital heart defects (25%−40%), urogenital anomalies (18%), musculoskeletal anomalies (16%) and central nervous system anomalies (10%) (3, 4). Despite medical and surgical advances, CDH continues to have high mortality and morbidity rates (5). Pulmonary hypoplasia and pulmonary hypertension (PH) are hallmarks of CDH presentation, resulting from both pulmonary vasculature and respiratory maldevelopment, the severity of which determine outcomes. In addition to these factors, there is also an increasing appreciation of early postnatal cardiac dysfunction as a determinant of outcome in CDH and use of agents to ameliorate cardiac dysfunction (6, 7).

Prostaglandin E1 (PGE1), a prostaglandin analog administered by intravenous infusion, is typically used to maintain ductal patency in newborn infants in the setting of suspected duct-dependent congenital heart disease. In this setting, PGE1 infusion is used to ensure adequate systemic or pulmonary blood flow or mixing until definitive surgical repair is accomplished. In the setting of pulmonary hypertensive disease, including CDH, PGE1 may have other benefits, the most important being reduction of afterload of a failing right ventricle (RV).

In this review, we will discuss the pathophysiology of CDH, the theoretical benefits of PGE1 therapy in the management of CDH, critically review existing evidence of its use, and identify key questions for future areas of research.

## Pathophysiology of CDH

Congenital diaphragmatic hernia is associated with pulmonary hypoplasia of varying extent typically affecting both the ipsilateral and contralateral lungs. This abnormal pathophysiology has been hypothesized to begin at ~8th to 10th-week of gestation, after failure of the normal physiological closure of the diaphragm and the establishment of the separation between abdominal and thoracic organs (8). A 2-hit hypothesis has been proposed to explain this spectrum of pulmonary hypoplasia. Following the initial “hit”, possibly genetic or environmental, during the early stages of organ development, bilateral lung hypoplasia occurs. This is followed by the second “hit” - compression of the ipsilateral lung by the hernia itself (9). Accompanying these changes in lung architecture are changes in the pulmonary vasculature, specifically early maturation, underdevelopment and increased muscularization of the pulmonary arterial vessels leading to altered vessel tone and reduced vessel caliber (10). Molecular pathways that are implicated in pulmonary vascular remodeling in CDH, which have been studied in humans and nitrofen rat models (11), include the retinol pathway (12), vascular endothelial growth factor (VEGF) (13), endothelin (14), Bone Morphogenic Protein (BMP) and Apelin (15). Alternations in these pathways may affect endothelial cell function, molecular signaling to the pulmonary arterial smooth muscle cells contributing to pulmonary arterial smooth muscle cell proliferation, and the characteristically hypertrophic pulmonary arterioles found in CDH-associated PH (CDH-PH) (6, 16).

The pulmonary alveolar and vascular maldevelopment results in increased pulmonary vascular resistance and associated PH. Studies have shown that over 70% of CDH infants exhibit CDH-PH (17), which is independently associated with increased mortality risk, oxygen support at 30 days, and utilization of extracorporeal life support (ECLS) (18). PH manifests as hypoxia due to right-to-left shunting across the atria, patent ductus arteriosus and any ventricular septal defect, if present, as well as increased afterload on the RV. In response to the increased afterload, the RV exhibits an initial adaptive dilatory response that may be followed by maladaptive hypertrophy and subsequent failure, which may be exacerbated by a restrictive ductus arteriosus. This RV failure may in turn result in impairment of diastolic filling of the left ventricle (LV) and reduced systemic blood flow. Myocardial ischemia of the RV plays an important role in the pathophysiology of cardiac failure in PH; specifically, through compromised right coronary blood flow. In PH, the right coronary perfusion gradient may be reduced due to the sustained increase in RV pressures and decrease in the aortic pressures (due to reduced LV preload and cardiac output) (19). Decreased RV coronary perfusion in the context of increasing myocardial oxygen consumption may predispose the RV to ischemia and dysfunction (20).

In addition, CDH has been described to be associated with both structural and functional left ventricular abnormalities (7, 21, 22). Fetal LV hypoplasia is well-described and possibly occurs due to mechanical compression, reduced fetal LV blood flow from reduced pulmonary venous return and altered streaming of venous return due to mediastinal shift (23–25). In a LV that may already be relatively hypoplastic due to the aforementioned reasons, the increase in afterload during the transition at birth, combined with the interdependent impacts of RV dilatation and dysfunction, can lead to significant LV dysfunction with adverse cardiopulmonary and hemodynamic outcomes (26). In a recent study, Patel et al. reported that early LV systolic function correlated with prenatal and postnatal markers of clinical disease severity (27). This observation underscores the importance of appropriate management of early PH in order to prevent biventricular dysfunction and associated impairment of systemic blood flow and oxygen delivery.

The combination and spectrum of pulmonary hypoplasia, pulmonary hypertension, and ventricular dysfunction makes CDH a unique clinical management challenge. Historically, PH therapeutic strategies in CDH have focused mainly on pulmonary vasodilation. The main therapeutic targets are cytokine pathways regulating pulmonary artery smooth muscle tone, specifically such the nitric oxide (NO) pathway, prostacyclin pathway and endothelin pathways (28).

Inhaled nitric oxide (iNO), a potent pulmonary vasodilator, acts by stimulating guanylyl cyclase in the vascular smooth muscle cells to produce cyclic guanosine monophosphate (cGMP). Elevated intracellular concentrations of cGMP activate cGMP-dependent protein kinases and lower cytosolic calcium concentrations, which in turn promote vascular smooth muscle cell relaxation (29). Phosphodiesterases (PDEs) are a large family of enzymes that hydrolyze cyclic nucleotides (cGMP and cAMP). Inhibition of PDEs leads to vasodilator effects. PDE5 (a cGMP-specific PDE), PDE3 and 4 (which hydrolyze cAMP) are expressed in the lung (28). Sildenafil, a PDE5 inhibitor that acts *via* the NO pathway, has been widely used in the management of CDH-PH (30). Milrinone, a PDE3 inhibitor, has also been studied in the CDH population (28, 31). Another important pulmonary vasodilator is prostacyclin; agents targeting this pathway include epoprostenol and inhaled iloprost (28, 32, 33). Inhibition of PDE3 causes lower pulmonary arterial pressures by acting *via* the PGI2 pathway (28). Endothelin (ET)-1 is a potent vasoconstrictor, and, hence, a target for modulating pulmonary vascular resistance. In a randomized control trial comparing the use of Bosentan, a drug which acts on the ETA and ETB receptors, with placebo as treatment for neonates with persistent pulmonary hypertension (PPHN), Mohamed et al. reported that Bosentan was superior to placebo for the treatment of PPHN (34).

## The RV in PH Disease: Animal Models

Among studies in adults with PH, the major cause of mortality of patients with PH was RV failure (35). Experimental animal models to investigate the effect of pressure overload on the RV include the monocrotaline (MCT) and chronic hypoxic mouse models (36) and the pulmonary artery banding (PAB) mouse model (37) which was developed to study the RV-specific effects independent of the pulmonary circulation. RV failure molecular mechanisms involve abnormal metabolism, impaired angiogenesis, mitochondrial dysfunction and increased oxidative stress (38–40). Further, the “sick lung circulation” hypothesis postulates that altered lung vascular cells from the “sick lung”, such as those containing cell fragments, free DNA and microRNA, can be cytotoxic to the RV and can re-program endothelial cell genes, thus contributing to the RV failure (40).

Several important concepts regarding ventricular response to elevated pulmonary pressures investigated in animal models can be translated to clinical medicine. Firstly, as demonstrated by Urashima et al. (41), the RV and LV do not respond identically to pressure overload; thus, treatment strategies that focus individually and specifically to each ventricle are important. Additionally, it has been shown that acute RV pressure overload impairs LV function by altering septal strain and apical rotation (42). The RV's molecular adaptation varies based on the degree of pressure overload as well as the type of pressure overload (proximal type as seen in the PAB model vs peripheral type seen in PH models vs combined pressure and volume overload as in the presence of a shunt) (43, 44). Severe PH can result not only in systolic, but also diastolic dysfunction (45). Hemodynamic measurements of the RV in response to PH have been shown to correlate and predict biomechanical changes in the myocardium (46). Pressure overload on the RV significantly alters the pressure-volume relationship, leading to greater end-diastolic pressures, and concurrently increasing the longitudinal elastic modulus [Elastic modulus (E) or the amount of force required to deform a tissue] in the PAB rat model (46).

Existing studies of RV function in CDH, though limited, indicate similar morphological and functional changes. Echocardiographic studies of early RV function have demonstrated RV systolic and diastolic dysfunction, and evidence of interdependent impairment of LV function (47, 48). Furthermore, early RV dysfunction pre- and post CDH repair have been shown to be associated with adverse outcomes, including increased mortality, ECLS use and length of hospitalization in survivors, in single center cohorts as well as large registry-based analyses (49–51).

An important conclusion that can be drawn from these animal model studies and clinical studies in CDH is the importance of unloading the RV in the setting of elevated pulmonary pressures, and tailoring PH therapy to target both biomechanical and hemodynamic function with the aim of optimizing RV function and improving outcomes.

## Role OF PGE1 in CDH

Prostaglandin E1 is a potent dilator of the ductus arteriosus in human neonates (52). The first studies ex-vivo in fetal lambs in 1973 by Coceani and Olley (53) led to clinical trials (54, 55) and approval for use by Food and Drug Administration (FDA) in 1981 (56).

**Figure 1:**
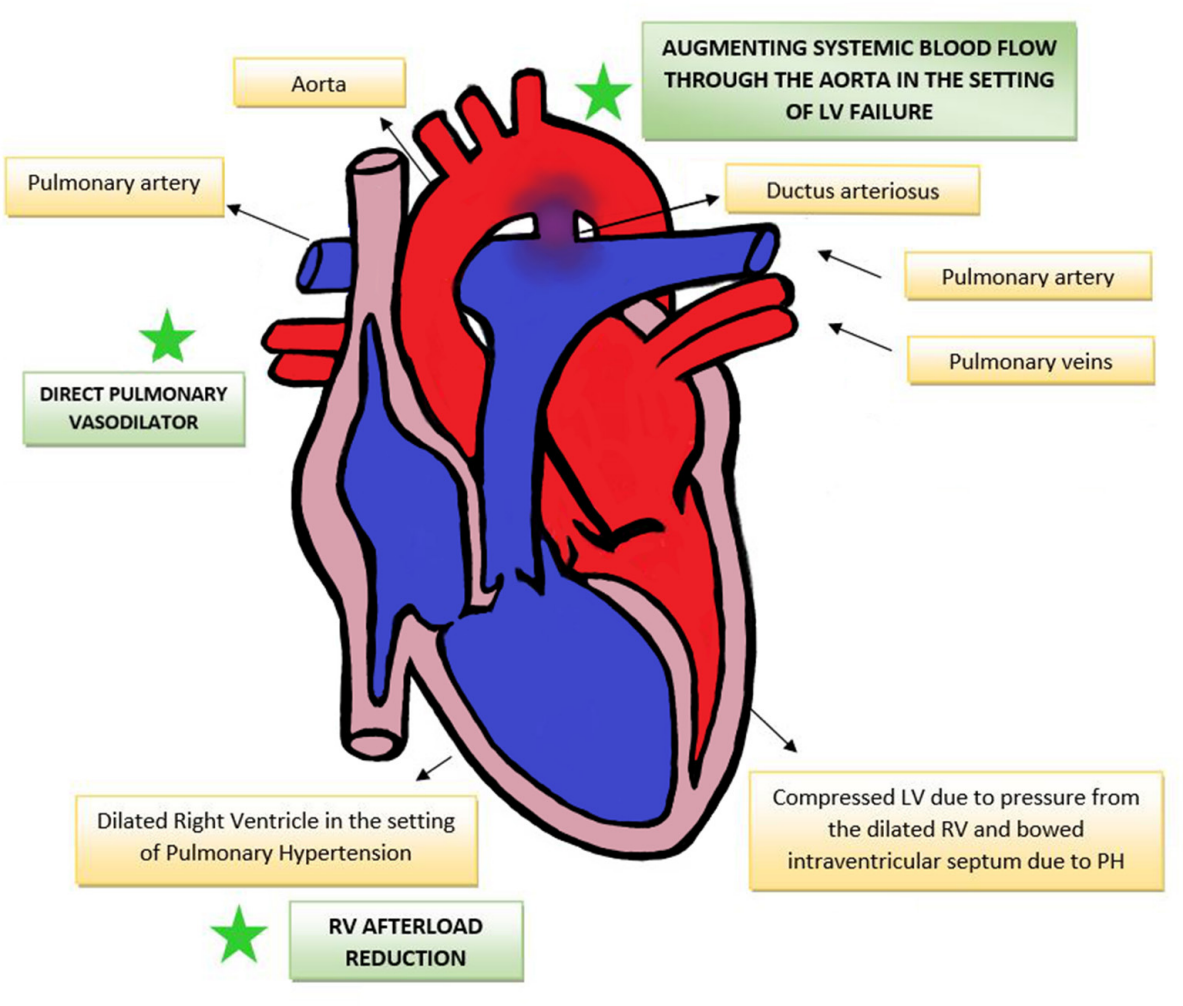
Effect of PGE1 in PH-RV afterload reduction, direct pulmonary vasodilation and augmenting systemic blood flow. RV, right ventricle; LV, left ventricle; PH, pulmonary hypertension.

The therapeutic benefits that PGE 1 offers in the setting of elevated pulmonary vascular resistance in CDH are theoretically three-fold. This has been summarized in Figure 1.

1) *By acting as pressure “blow-off” valve, reducing the effective afterload on the pressure loaded RV, alleviating RV dilatation and myocardial dysfunction*. LV function in turn may also improve by mechanisms of ventricular interdependence. A similar strategy of having a “pop off” conduit in supra-systemic pulmonary pressures has been demonstrated by the use of the Pott's shunt (anastomosis between left pulmonary artery to descending aorta) in pediatric hypertension and in patients with Eisenmenger syndrome (57). Evidence from pediatric patients with pulmonary hypertension have shown that a Pott's shunt improves RV-systolic function and RV-PA coupling, resulting in overall improved functional status and transplant-free survival (58).
2) *By augmenting systemic blood flow in the setting of LV failure, by facilitating right-to-left shunting via the ductus arteriosus*. The evidence of the benefits of using PGE1 to augment systemic blood flow is best noted in single ventricle pathologies such as hypoplastic left heart syndrome, where there exists an uncertain balance among systemic, pulmonary and coronary blood flows, with the systemic and pulmonary circulations in parallel rather than in series. The use of PGE1 in this situation ensures systemic blood flow to the vital organs, and also balances the systemic and pulmonary cardiac output (59).
3) *By its direct pulmonary vasodilating action in pulmonary artery smooth muscle*. PGE1 increases intracellular cyclic AMP leading to decreased pulmonary vascular resistance (60), reducing RV afterload and potentially improving coronary perfusion to the RV (20). The pulmonary vasodilator benefits of PGE1 in primary pediatric pulmonary hypertension (61) and in neonatal PPHN have been demonstrated previously (62, 63).

Animal studies support these potential benefits. Sakuma et al. (64), demonstrated in a monocrotaline rat PH model that PGE1 administration significantly reduced the production of cytokines IL-1, IL-6 and TNF, previously implicated in pulmonary hypertension. In another study by Ono et al. (65), PGE1 had a dose-dependent suppression of RV hypertrophy and pulmonary hypertension in a MCT rat model.

Though the potential benefits of PGE1 use in the cardiopulmonary physiology of CDH appear compelling, there are potential adverse effects. In the short-term, PGE1 may induce apnea, peripheral vasodilation, fever and hypotension (66). With long term use (>5 days), cortical hyperostosis, brown fat necrosis, gastric outlet obstruction and intimal mucosal damage have been reported (66). Worsening hypoxia due to right-to left ductal shunting should also be considered (67).

## Review of Clinical Studies

To date, the investigation of PGE1 use in CDH has been limited to case reports and retrospective chart reviews. We performed a review of literature using the electronic bibliographic databases PubMed and Embase, and of ongoing trials in www.clinicaltrials.gov. Additionally, we also used PubMed's related citations feature to identify relevant studies. We included chart reviews, case control studies, case series and case reports. Once a list of studies was obtained, we analyzed the studies for methodology and outcomes measures as described below.

A summary of these studies is provided in Table 1.

**Table 1:** Summary of clinical studies on PGE1 use in CDH.

**Author**	**Year of publication &** **country**	**Study design**	**n**	**Indications for PGE1**	**Age at use**	**Dosing of PGE**	**Duration of therapy (Days)**	**Echocardiographic assessment**	**Outcome measures**	**Results/outcome of the study**
Inamura et al. (68)	2005 (Japan)	Retrospective review	Total-19; PGE (+) 9	Duration of the R-L shunt through the DA was longer than that of L–R shunt	–	3–5 ng/kg/min	-	1. LV diastolic diameter index [LVDI] 2. Total pulmonary artery index [TPAI] 3. LV-Tei index Measured on DOL 0 & 2	Echocardiographic markers of LV dysfunction	1. LVDI and TPAI of day 0 in PG (+) were significantly smaller 2. LV Tei index on postnatal day 0 in PG (+) was significantly higher
Shiyanagi et al. (23)	2008 (Japan)	Retrospective review	PG+iNO-19, iNO-30	Echo signs of PH	–	0.05–0.20 μg/kg/min	–	1. Dominant R-L shunt through a PDA 2. Decrease in pulmonary arterial blood flow on the affected side. 3. Tricuspid regurgitation velocity (TRV) > 2.5 m/s.	1. Survival rate 2. Length of hospital stay 3. Timing of surgical repair 4. Timing of spontaneous close of DA	1. No significant difference between survival rates between the groups 2. Hospital stay was significantly shorter in the iNO group 3. Earlier surgery in iNO group 4. Spontaneous closure of PDA was early in iNO group
Inamura et al. (69)	2014 (Japan)	Retrospective review	Total-61 PGE (+)39 PGE (–) 22	Duration of the R-L shunt through the DA was longer than that of L–R shunt	–	3–5 ng/kg/min	–	1. The LV end-diastolic diameter, corrected for body surface area (LVDD/BSA) 2. Ejection fraction (EF), 3. Tei index-LV	Echocardiographic markers of LV dysfunction	1. Improved LV function shown by significant increase in LVDD and LV-Tei index
										
Lawrence et al. (70)	2019 (USA)	Retrospective review	PGE (+)57	1) Echo findings of PH with a restrictive PDA 2) Persistent metabolic acidosis (pH <7.25 with base deficit or lactate elevation) without LV dysfunction on echo, or 3) Post ductal arterial oxygen content <30 mmHg.	DOL 9 (IQR 2–13)	0.01–0.05 μg/kg/min	17 ± 2	1. TR jet velocity 2. DA direction 3. Septal position	1. BNP levels 2. Echo markers of severe PH	1. BNP levels declined after 1.4 ± 0.2 days and again at 5.2 ± 0.6 days after treatment 2. Echo markers of severe PH improved significantly, after 6 ± 0.8 days of treatment
Le Duc et al. (26)	2020 (France)	Retrospective review	PGE (+)-18	Maximal R-L blood flow velocities are > 1.5m/s with acute worsening of the cardiorespiratory status	DOL 11 (IQR 5–16)	0.025–0.05 μg/kg/min	3	1. Maximal blood flow velocities and flow patterns through the DA 2. Mean PAP compared to the systolic blood pressure measured on echo and classified as supra-systemic when mean PAP is ≥ systemic blood pressure + 10 mmHg	1. Decrease in FiO_2_ 2. Ductal flow direction and velocities	1. Significant decrease in FiO_2_ at hour 6 (median FiO_2_ decreased from 80% to 34% to target preductal SpO_2_ between 88% and 96%) 2. Significant decrease in maximal blood flow velocities in the DA

*PGE1, Prostaglandin E1; R-L, Right to left; L–R, Left to Right; DA, Ductus Arteriosus; RV-right ventricle; LV, Left Ventricle; iNO, Inhaled Nitric Oxide; PDA, Patent Ductus Arteriosus; PH, Pulmonary Hypertension; PAP-Pulmonary Arterial Pressure; BNP, Brain Natriuretic Peptide; DOL, Day of Life*.

### Methodology and Indications of PGE1 Use

All but one of the studies of PGE1 use in CDH-PH have been retrospective chart reviews, comparing patients who received PGE1 with those who did not (26, 68–70), one retrospective study compared the combined therapy of PGE+iNO with those only receiving iNO (23). Three studies reported initiating PGE1 based only on echocardiographic parameters. Inamura et al. (68, 69) reported initiating PGE1 infusion when the duration of right-to-left shunting *via* the DA was longer than that of left-to-right shunting, whereas Shiyanagi et al. (23) reported using PGE1 for PH based on echocardiographic signs: dominant right-to-left shunt through a PDA, decrease in pulmonary arterial blood flow on the affected side, and tricuspid regurgitation velocity (TRV) more than 2.5 m/s. Two of the studies reported using PGE1 based on a specific criterion; Lawrence et al. delineated specific indications for PGE1 initiation based on institutional CDH guidelines, which included (1) echocardiographic findings of PH with a restrictive PDA, (2) persistent metabolic acidosis (pH <7.25 with base deficit or elevation of lactate) without left heart dysfunction on echocardiography, or (3) persistent post-ductal arterial oxygen content <30 mmHg (70). In the study by Le Duc et al. (26), PGE1 was initiated when the maximal right-to-left blood flow velocities were >1.5m/s in CDH infants with acute worsening of the cardiorespiratory status.

As noted, most of the cited studies specified a right-to-left ductal shunting pattern as an indication for initiation of PGE1. One study mentioned a “restrictive PDA” as a criterion, but no specific duct size measurement was reported (70). None of the studies report echocardiographic evidence of abnormal RV size or function as a marker for PGE1 initiation, although clinical signs of RV/LV failure were used as criteria in two of the cited studies (26, 70). One study used plasma BNP before and after PGE1 initiation as a measure of PH and RV strain, and demonstrated decline in BNP measurements and improvement in echocardiographic measures PH (70). Plasma BNP peptides are secreted in response to wall stress by both the ventricles. However, a study by Koch et al. demonstrated the rapid decrease in plasma BNP levels during the first week of life, and the use of plasma BNP as a marker of clinical improvement may not necessarily reflect the effect of PGE1 use (71).

### Outcome Measures to Assess Response to PGE1

Echocardiographic markers have been used to assess the effect of PGE1: two studies used LV size and function (measuring LV diastolic diameter, total pulmonary artery index (TPAI), left ventricular end-diastolic diameter and LV-Tei index (a composite measure of LV function based on systolic and diastolic time intervals) (68, 69). One study used echocardiographic markers of PH (estimated RV systolic pressure using tricuspid regurgitation jet velocity, direction of flow across a patent ductus arteriosus, and ventricular septal position) (70), and one study reported ductal flow direction and velocities (26).

In the studies by Inamura et al. (68, 69), the authors concluded that in instances of severe PH keeping the ductus open plays an important role in the circulatory management of these patients by improving LV function (as indicated by a higher LV Tei index in infants receiving PGE1). Lawrence et al. (70) observed that use of PGE1 significantly reduced B-Natriuretic peptide levels (BNP, a plasma biomarker of pulmonary hypertension and associated cardiac strain) and echocardiographic indices of PH, as assessed by tricuspid regurgitation jet velocity, ductus arteriosus direction, and ventricular septum position. Le Duc et al. (26) concluded that use of PGE1 in CDH decreased FiO_2_ requirements (median FiO_2_ decreased from 80% to 34% to target preductal SpO_2_ between 88 and 96%), and improved circulatory function, thus preventing cardiorespiratory failure in this population. Echocardiographic markers for PH used in this study include ductal flow velocities and flow patterns and mean pulmonary arterial pressures in relation to systemic blood pressures and classified as suprasystemic when mean PAP > systemic blood pressure +10 mm Hg (26). An observational study conducted by Hofmann et al. (72) reported that use of PGE1 in addition to circulatory management with catecholamines in two of their patients with CDH-PH relieved and stabilized right ventricular function. Although these studies reported on common echocardiographic markers of PH and LV dysfunction parameters, it is notable that none of the cited studies assessed RV function.

Most of the studies report improvement in cardiopulmonary outcomes with the use of PGE1 in CDH. However, a retrospective study by Shiyanagi et al. (23) demonstrated no significant clinical effects with the use of PGE1 combined with iNO, and concluded that use of iNO alone would simplify the management of PH due to CDH. The study reported no significant difference in survival to discharge, however, a shorter duration of hospitalization and earlier dates of repair were observed for those receiving iNO alone (23). Interestingly, this was also the only study to report PDA diameter, timing of spontaneous PDA closure and other long-term outcomes such as length of stay and survival to discharge (23).

In terms of adverse effects of PGE1, Shiyanagi et al. (23) reported lower systemic BP in the group that received PGE1 in comparison to those that did not. Lawrence et al. (70) reported seven patients with side-effects due to PGE1 which included pulmonary overcirculation due to L–R shunting (2%), cortical proliferation of their long bones (5%), temperature elevation (1.8%) and GI bleed (1.8%). However, none of the studies reported any life-threatening adverse effects or mortality attributed to the use of the medication.

In addition to the above studies, case reports describing improvement of cardio-respiratory function following use of PGE1 infusion are summarized in Table 2 (73–76).

**Table 2:** Summary of case reports on PGE1 use in CDH.

**Author**	**Year of publication & country**	**Patient description**	**Indications**	**Age at use (DOL)**	**Dosing of PGE**	**Duration of therapy**	**Echocardiographic assessment after PGE1 use and at discharge**	**Discharge (DOL)**	**PH therapy at discharge**
Buss et al. (73)	2006 (Australia)	GA-41 weeks BW-3,094 g	DOL8 Echo- 1) Suprasystemic PAP 2) Severe TR with a pressure gradient in excess of 100 mm Hg and 3) Near complete closure of DA 4) Clinical signs of RV failure	8	10 ng/kg/min	20 days	**After PGE1**-Good RV function with only mild tricuspid valve regurgitation and reversion to bidirectional shunting across the PDA **At Discharge**- spontaneous closure of the duct and a fall of PAP to half systemic levels, mild tricuspid valve regurgitation. Good RV function	69	None
Filan et al. (74)	2006 (Australia)	GA-34 weeks BW-1,907 g	DOL 12- Echo- 1) Severe PH (based on TR jet) and right heart failure, 2) Systolic RV pressures equal to twice systemic, 3) Ventricular septum was bowing into LV cavity, 4) Closed DA	12	10 ng/kg/min	8 days	**After PGE1-**Ductal patency, reduction of RV pressures and improved RV function **At Discharge-**Good right ventricular function, a closed duct, and L–R interatrial shunting	54	
Divekar et al. (75)	2015 (USA)	GA- Full term BW-4,000 g	DOL1- Echo- 1) Restrictive PDA with R-L shunt, 2) Severe TR predicting supra-systemic PAP (TR 95 mm Hg, SBP 55/40 mm Hg), 3) Severe right ventricular (RV) dilation with septal bulge into the left ventricle (LV), and 4) Moderately reduced RV systolic function	1	0.05 mcg/kg/min	32 hours	**After PGE1**-Non-restrictive PDA with right to left shunting, improved RV systolic function, reduction in severity of TR, reduction in PAP from supra-systemic to systemic (TR 65 mm Hg, SBP 65/45 mm Hg), decreased septal shift (less LV compression) **At Discharge**- sub-systemic PAP	14	Sildenafil
Aljohani et al. (76)	2020 (USA)	GA-full term BW-3,280 g Prenatal markers- O/E TFLV 25%	DOL9-Echo- 1) RV pressure >2 × systemic pressure based on TR jet 2) Septal motion, 3) Small PDA with R-L shunt, 4) Diminished RV function	9	-	41 days	**After PGE1-** Reduction in RV systolic pressures **At Discharge-**Normal septal position and RV size	78	Sildenafil Bosentan

*GA, gestational age; BW, birth weight; DOL, day of life; TR, tricuspid regurgitation; PAP, pulmonary arterial pressure; PGE1, prostaglandin E1; R-L, right to left; L–R, Left to Right; DA, Ductus Arteriosus; RV-right ventricle; LV, Left Ventricle; iNO, Inhaled Nitric Oxide; PDA-Patent Ductus Arteriosus; PH-Pulmonary Hypertension; PAP-Pulmonary Arterial Pressure; O/E TFLV-Observed to Expected Total Fetal Lung Volume*.

The above-described retrospective clinical studies and case reports indicate a potential cardio-respiratory benefit in using PGE1 in the managing PH in the CDH population. Why then is PGE1 not a routine component of the management of patients with CDH? (77, 78). Likely factors include uncertainty in identifying the appropriate subset of patients with CDH-PH, who might potentially benefit from this approach, and possibly the concerns relating to the short-term adverse effects and/or long-term adverse effects of having the ductus open. Possible ways to address these relevant concerns are two-fold:

1) Advocating for a more pronounced pathophysiology-based approach using serial echocardiograms.
2) Promoting further research on the use of PGE1 in this patient population to address these clinical concerns.

To our knowledge, there have not been any prospective studies investigating the effect of PGE1 on cardiorespiratory outcomes in CDH. Studies on significant and/or long-term outcomes, such as need for ECMO, duration of ventilation, length of stay, need for oxygen at discharge, need for additional PH medications and neurodevelopmental outcomes, are lacking. Use of PGE1 infusion either alone or in combination with other PH management strategies is a potential area for future research, which may further open the doors to other aspects of research such as the long-term effects of the presence of a ductus in patients with congenital diaphragmatic hernia.

## Future Investigations of Pge1 Infusion in CDH

Ongoing areas of uncertainty about the use of PGE1 in CDH which require further investigation include:

1) Appropriate timing of the PGE1 infusion in CDH (e.g., earlier prophylactic administration vs. later after echocardiographic evidence of PH).
2) Dosing regimens of PGE1 for PH (e.g., fixed dosing or dose titration based on clinical and echocardiographic response). The studies described above have used variable dosing patterns for PGE1 use in CDH. Although this area may need to be further investigated, higher doses of PGE1 may have utility in situations of acute severe PH with RV failure with duct closure, analogous to those used in resuscitation of infants presenting with duct closure in critical congenital heart disease.
3) Potential side effects of its use in this population (both short- and long-term).
4) Duration of therapy (e.g., fixed or based on clinical and echocardiographic response).
5) Use of PGE1 in relation to ECLS.
6) Impact of concomitant use of other pulmonary vasodilators, such as iNO, Sildenafil and/or Bosentan.
7) Impact on LV/RV performance.
8) The specific subset of CDH that might best benefit from it use, which may require a pathophysiology-based, targeted approach to PH management in CDH.

In terms of “long-term” effects, areas of uncertainty ripe for further investigation include:

1) Impact on short- and long-term outcomes, including survival, need for ECLS, duration of ventilation, duration of hospital stay, need for oxygen at discharge, and neurodevelopmental outcome.
2) Timing and need for additional PH medications, such as Sildenafil and/or Bosentan at discharge.
3) Impact on intervention for ductal management due to the potential effect from L–R shunting.

With the evidence from a recent study that the severity of early postnatal PH has a significant impact on long-term outcome, an important question to be answered is the timing of the first echocardiogram in this population (18). This study stressed the importance of an early echocardiogram as a valuable prognostic tool that could potentially provide information that can impact the clinical course and management of PH.

Theoretically a well-designed clinical trial of PGE1 in CDH may help to address the current evidence gap. Ideally this would be a randomized double-blinded placebo trial of PGE1 in a priori risk-stratified subgroups of CDH patients with echocardiographic and clinical evidence of elevated PAP, biventricular dysfunction and a restrictive ductus and with outcome measures that include cardiopulmonary outcomes such as RV and LV performance, need for ECLS, effect of ventilatory needs, vasopressor needs, survival at discharge and mortality, managed using standardized management guidelines. Risk stratification should include prenatal imaging markers of severity, such as percent liver herniation (%LH), observed to expected Total Fetal Lung Volume (O/E TFLV), observed to expected Lung-Head Ratio (O/E LHR) (79), location of birth (in-born vs. out-born patients), etc.

Previous milestone trials in CDH include Ventilation in Infants with Congenital diaphragmatic hernia (VICI), the TOTAL trial of fetal tracheal occlusion, and the Neonatal Inhaled Nitric Oxide Study (NINOS) (80–82). Others are in progress include Congenital Diaphragmatic hernia Nitric Oxide vs. Sildenafil (CoDiNOS) trial and a randomized pilot trial of milrinone in congenital diaphragmatic hernia (31, 83). However, study recruitment for well-powered trials is a common challenge, which unfortunately can lead to smaller pilot studies without adequate power (84). Research in the CDH population is challenging due to small number of patients with isolated CDH and lack of evidence-based treatment strategies. Registry-based studies may be useful, but the wide variability in CDH management amongst institutions within the registry limit researchers' ability to draw meaningful conclusions or extrapolate the results to clinical practice (84). Investigating a therapeutic strategy of the use of PGE1 in patients with CDH, based on their clinical markers and echocardiographic indices of RV/LV dysfunction, could be thought of as a “pathophysiology-based approach” toward promoting precision medicine in this population. Such trials are sorely needed and will likely require multi-center collaboration to be completed in a timely fashion.

## Conclusion

In conclusion, with continuing research to improve cardio-pulmonary and long-term outcomes in CDH, new management strategies are being proposed and studied. Supra-systemic RV pressures are associated with poor clinical outcomes in this population. There is a pathophysiological rationale for the use of PGE1 in CDH to maintain ductal patency and promote right-to-left shunting, thereby reducing effective RV afterload and supporting systemic blood flow. In addition, PGE1 may have direct pulmonary vasodilating actions. Although existing, single-center retrospective studies and case reports suggest benefit from the use of PGE1 in terms of reducing severity of PH and improving short-term cardiopulmonary stability, uncertainties remain around its optimal pragmatic clinical use in CDH, and current evidence from these studies may not strongly support clinical recommendations. Conducting pharmacological trials in neonates can be challenging due to physiological changes, variable pharmacokinetics in the early newborn period and the ethical considerations involved. However, a well-designed a-priori prospective study as outlined above should be considered to definitively understand the implications of the use of PGE1 in CDH and its impact on meaningful outcomes.

## Author Contributions

SH: conceptualization, design, methodology, and drafting and revising. NP: design, methodology, and reviewing and revising. CF: conceptualization, design, reviewing and revising, and supervision. All authors contributed to manuscript revision, read, approved the submitted version and agreed to be accountable for all aspects of the work.

## Conflict of Interest

The authors declare that the research was conducted in the absence of any commercial or financial relationships that could be construed as a potential conflict of interest.

## Publisher's Note

All claims expressed in this article are solely those of the authors and do not necessarily represent those of their affiliated organizations, or those of the publisher, the editors and the reviewers. Any product that may be evaluated in this article, or claim that may be made by its manufacturer, is not guaranteed or endorsed by the publisher.
